# Interlayer‐Driven Interfacial Stabilization in Solid Electrolytes for Lithium Batteries: Promises and Challenges

**DOI:** 10.1002/cssc.70673

**Published:** 2026-05-06

**Authors:** Madan Bahadur Saud, Hansheng Li, M. Bilal Faheem, Ruosi Qiao, Yeqing Wang, Quinn Qiao

**Affiliations:** ^1^ Department of Mechanical and Aerospace Engineering Syracuse University Syracuse New York USA

**Keywords:** anode‐electrolyte interface, cathode‐electrolyte interface, interfacial engineering, interlayer engineering, solid electrolytes, solid‐state batteries

## Abstract

Despite major progress in developing solid electrolytes (SEs) with high ionic conductivity, the performance of all‐solid‐state Li‐metal batteries (ASSLBs) remains dominated by interfacial impedance that develops at the electrode‐electrolyte interface. Sulfide and halide SEs have emerged as leading candidates for high‐energy density ASSLBs owing to their exceptional ionic conductivities, low grain‐boundary resistance, and favorable mechanical deformability. However, their practical implementation is still constrained by severe interfacial instabilities with both Li‐metal anodes and high‐voltage layered oxide cathodes. Interlayer engineering, specifically the incorporation of a functional interlayer between the electrode and SE, has become one of the most effective strategies to mitigate these challenges, enabling suppression of electrolyte decomposition, reduction of space‐charge effects, homogenization of Li flux, and stabilization of interphases under high current densities. In this review, we aim to recapitulate the recent developments made in the interlayer‐engineering approaches that span over a range of sulfide‐ and halide‐based SE systems, which play a central role as fast Li^+^‐conducting media in enabling high‐energy‐density ASSLB architectures, and distill unified design principles that connect electrochemical stability, ion‐transport behavior, and mechanical compliance. Finally, we discuss future directions and research opportunities that define key priorities for scalable interlayer engineering, aimed at accelerating the development of next‐generation high‐performance ASSLBs.

## Graphical abstract

Interfacial instability severely limits sulfide and halide solid electrolytes in solid‐state lithium batteries. The interlayer engineering strategies that suppress dendrites, electrolyte reduction, space‐charge effects, and uneven CEI formation have crucial importance to address these issues. By introducing stable passivation layers and improved interfacial contact, these interlayers significantly enhance the electrochemical performance of solid‐state batteries.

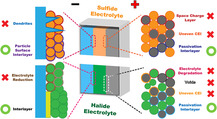

## 1 Introduction

Since their commercialization in 1991 by Sony Corporation, rechargeable lithium‐ion batteries (LIBs) have become indispensable in portable electronics, electric vehicles, and grid‐scale storage [1]. However, the liquid electrolytes used in conventional LIBs raised safety concerns that inherently limit further developments through energy density and cycle life, which have motivated significant global interest in all‐solid‐state batteries (ASSBs), which replace the flammable liquid electrolytes with solid‐state lithium‐ion‐conducting materials [2, 3, 4]. This substitution offers several key advantages, including enhanced intrinsic safety arising from the elimination of flammable organic liquid electrolytes and suppression of thermal runaway; higher electrochemical and thermal stability; wider operating temperature windows; and the possibility of pairing high‐capacity electrodes, such as lithium metal and high‐voltage transition‐metal oxides [5, 6, 7, 8]. As a result, ASSBs are widely regarded as a transformative pathway toward next‐generation energy storage systems with improved energy density and operational reliability [9, 10]. A central benefit of ASSBs is the potential use of Li metal anodes, which provides an ultrahigh theoretical capacity (3860 mAh g^−1^) and the lowest electrochemical potential (−3.04 V vs standard hydrogen electrode) among all anode materials reported to date [2, 9, 11, 12]. Substituting the conventional graphite anode (theoretical capacity ≈372 mAh g^−1^) with high‐capacity alternatives such as Li metal (≈3,860 mAh g^−1^) offers a substantial boost in energy density. At the electrode level, this transition increases the theoretical energy density from ∼468 Wh kg^−1^ for graphite‖LMO_2_ (*L* = Li; M = Ni, Co, or Mn) to approximately ∼701 Wh kg^−1^ for Li‖LMO_2_ configurations [13]. Traditional LIBs rely on graphite intercalation anodes largely because liquid electrolytes cannot stabilize the reactive Li surface; dendrite growth and continuous side reactions, such as electrolyte reductive decomposition, unstable solid–electrolyte interphase (SEI) formation, and parasitic reactions between lithium metal and electrolyte solvents/salts, lead to rapid cell failure [14, 15]. In contrast, appropriate solid electrolytes can physically impede dendrite penetration, form stable solid–solid interfaces, and operate at higher voltages without oxidative degradation. Moreover, ASSBs enable compact designs with minimized packaging and improved volumetric energy density—an important metric for electric vehicles and grid storage applications [9]. Among the families of solid electrolytes investigated, sulfide‐based and halide‐based materials have emerged as the two most promising candidates for practical ASSLB implementation. Sulfide SEs such as Li_10_GeP_2_S_12_ (LGPS), Li_7_P_3_S_11_ (LPS), and Li_6_PS_5_ *X* (LPS*X*, *X* = Cl, Br, I) exhibit high room‐temperature ionic conductivities comparable to or higher than those of liquid electrolytes and excellent deformability, along with low elastic moduli that facilitate intimate solid–solid interfacial contact [16, 17, 18]. More recently, lithium halide solid electrolytes (HSEs), often described by the general formula Li_3_MX_6_, where *M* represents group 13 metal elements, and *X* denotes one of the halogens (F, Cl, Br, or I), have emerged as an exciting alternative [19, 20, 21]. HSEs such as Li_3_YCl_6_, Li_3_InCl_6_, and their doped derivatives offer several attractive attributes, including high room‐temperature ionic conductivities and wide electrochemical oxidation limits approaching 4.5–5.0 V, enabling compatibility with Ni‐rich cathodes [20]. Also, HSEs exhibit ductility and relatively low grain‐boundary resistance, which allows densification at modest pressures.

**FIGURE 1:**
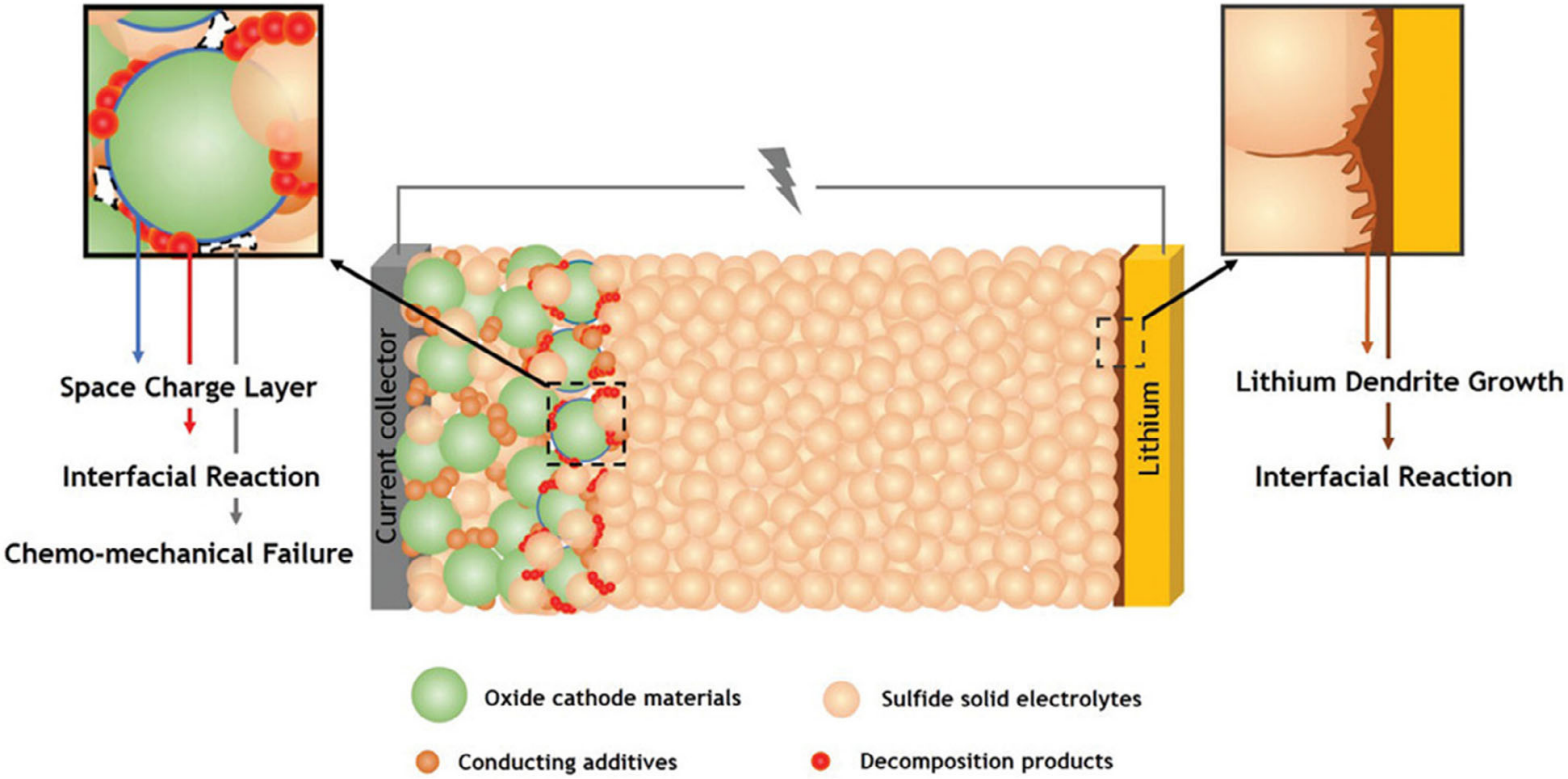
Schematic illustration of key interfacial degradation mechanisms in sulfide‐based ASSLBs. At the oxide cathode/sulfide solid electrolyte interface, space‐charge layer formation, interfacial chemical reactions, and chemo‐mechanical failure lead to contact loss and increased interfacial resistance, while at the Li metal/sulfide electrolyte interface, interfacial reactions and nonuniform Li deposition promote lithium dendrite growth and eventual short‐circuiting [22].

Despite their promise, the performance of ASSLBs has been impeded primarily by the unresolved challenges at two critical interfaces: (i) Li metal anode/SE interface and (ii) cathode/SE interface. At the Li metal/SE interface, interfacial reactions and nonuniform lithium deposition promote dendrite growth, ultimately causing short‐circuiting and cell failure, as depicted in Figure 1. The contact between Li metal and SE can be broadly categorized into three interfacial regimes [23]. In the first case—the thermodynamically stable interface—the solid electrolyte does not react with Li, yielding a sharp two‐phase boundary at equilibrium. Such systems are rare and require inherent chemical compatibility. More commonly, solid electrolytes react with Li to form a three‐dimensional interphase. When the reaction products exhibit both ionic and electronic conductivity, the interphase grows continuously into the electrolyte, forming a mixed‐conducting interphase (MCI). This “insertion‐type” interphase enables electron percolation, ultimately leading to electrolyte reduction and self‐discharge—representing an intrinsically unsuitable SE/Li pairing. Because MCI formation is a thermodynamically driven process, it can only be suppressed by introducing an artificial protection layer, often referred to as an artificial solid–electrolyte interphase (SEI). Alternatively, if the reaction products are electronically insulating, interphase growth becomes self‐limiting, resulting in a metastable but electronically blocking SEI. Like liquid‐electrolyte SEI layers, the battery performance then depends on the ionic transport properties of this thin passivation film. Sulfide SEs suffer from narrow electrochemical windows and reactive decomposition products, while HSEs undergo rapid reduction at Li metal, forming electronically conductive In^0^ or Y^0^ along with LiCl, as demonstrated through operando X‐ray photoelectron spectroscopy (XPS) and impedance spectroscopy [24, 25]. While the aforementioned discussion highlights instability at the Li metal/SE interface for both sulfide and halide SEs, interfacial challenges at the cathode side remain equally critical, particularly for sulfide‐based systems operating at high voltages. As shown in Figure 1, at cathode/SE interface, sulfide SEs paired with high‐voltage layered oxide cathodes (e.g.,Lithium nickel manganese cobalt oxide, LiNi_1–*x*–*y*_Mn_*x*_Co_*y*_O_2_, where *x* and *y* represent the molar fractions of Mn and Co, respectively, acronymic NMC and Lithium cobalt oxide, LiCoO_2_ acronymic LCO) suffer from space‐charge layer formation, interfacial reactions driven by oxidative electrolyte decomposition, and chemo‐mechanical contact loss, which collectively lead to severe interfacial impedance growth and degraded long‐term cycling performance in sulfide‐based ASSLBs [26, 27, 28]. Protective interlayers, compositional engineering, and tailored cathode coatings have been developed to mitigate these issues, but long‐term stability remains a major bottleneck. In this review, we critically examine and critique the key degradation pathways that undermine electrode/SE interfacial stability, summarize recent advances in interlayer strategies for interface stabilization, and highlight emerging interlayer design principles and research directions needed to enable the reliable integration of sulfide and halide SEs into next‐generation ASSLBs.

## 2 Engineering of Li Metal Anode/Sulfide SE Interfaces

**FIGURE 2:**
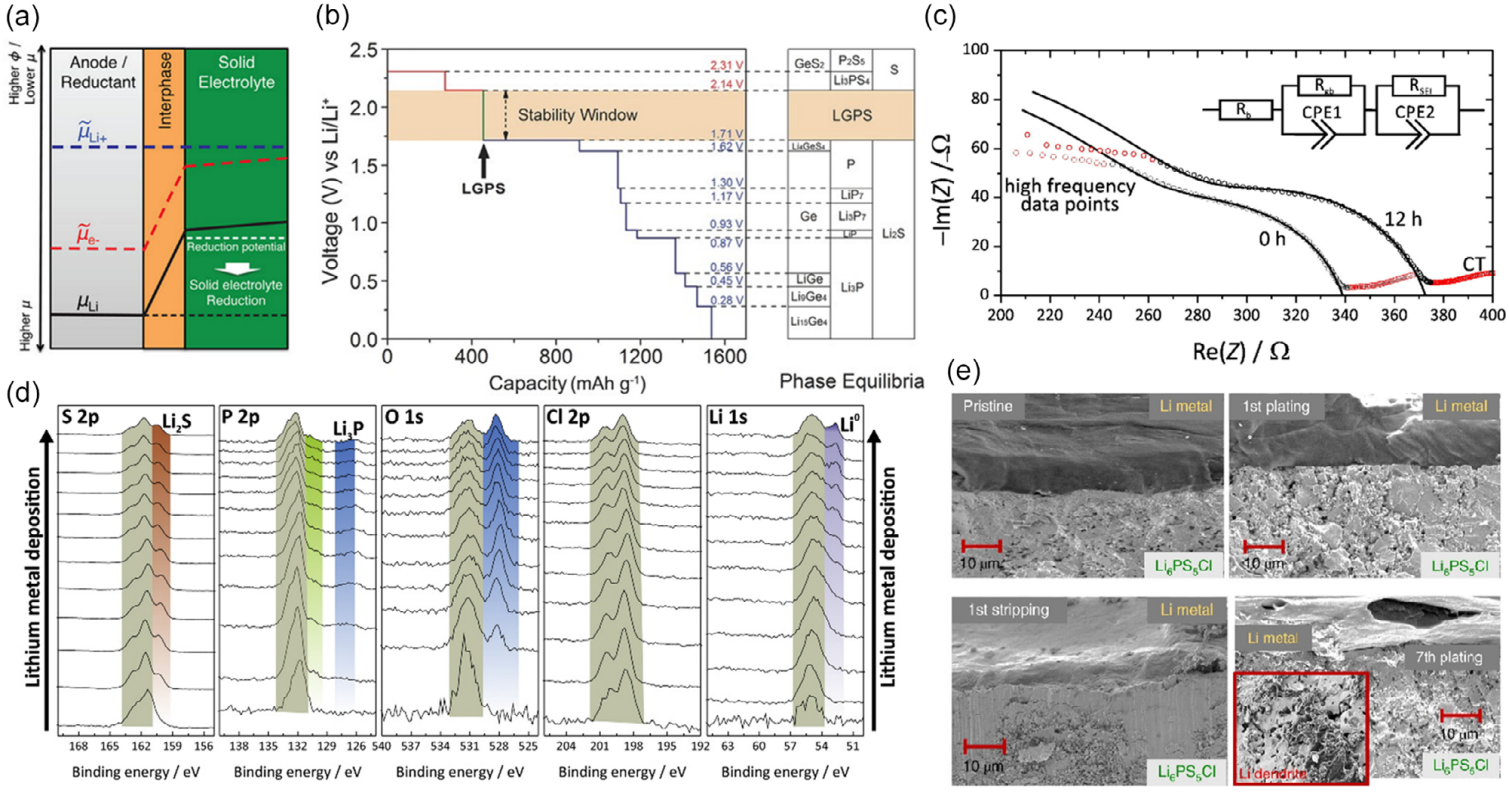
Interphase formation and electrochemical degradation at the Li metal, sulfide SE interface. (a) Schematic illustration of the thermodynamic and electronic driving forces at the Li metal/sulfide solid electrolyte interface [29]. (b) Calculated thermodynamic stability window of LGPS‐type sulfide electrolytes based on phase‐equilibria analysis [31]. (c) Time‐dependent Nyquist plots of a symmetric Li/LPS/Li cell showing the evolution of interfacial impedance upon contact with Li metal [25]. (d) S 2p, P 2p, O 1s, and Cl 2p XPS spectra of an LPSCl collected during Li deposition, revealing the formation of Li_2_S‐ and Li_3_P‐rich interphase species [33]. (e) Cross‐sectional SEM images of the Li/LPSCl interface at different stages of Li plating and stripping, illustrating contact evolution, void formation during stripping, and filament penetration into the sulfide electrolyte upon subsequent plating [36].

Significant interfacial challenges, including inadequate physical contact, limited chemical and electrochemical stability, dendritic lithium growth, and the buildup of mechanical stress at the Li/sulfide SEs interface, continue to hinder long‐term cycling stability of sulfide‐based ASSLBs [29, 30]. A primary origin of these failures is the poor thermodynamic stability of sulfide solid electrolytes against Li metal [31]. Several computational and experimental studies revealed that most of the highly conductive sulfide‐type solid electrolytes, such as LGPS, LPS, and Li_6_PS_5_Cl (LPSCl), undergo reduction at low potentials and oxidation at high potentials, despite their originally reported wide electrochemical stability windows derived from cyclic voltammetry (CV) [31, 32, 33]. First‐principles thermodynamic analyses show that sulfide SEs possess intrinsically narrow electrochemical stability windows, with thio‐phosphate SEs reducing at ∼1.6–1.7 V and oxidizing near ∼2.0–2.3 V, demonstrating that, despite their widespread use, they are not intrinsically stable against either Li metal or high‐voltage cathodes [31]. Although cyclic voltammetry often suggests a wider stability window, this apparent stability is now understood to arise from kinetic limitations and overpotential‐driven suppression of decomposition, and current understanding suggests that decomposition products form passivating interphases that slow further electrolyte degradation and create the appearance of a wider operating window [33, 34, 35]. Fundamentally, the thermodynamic driving force for chemical reactions at the electrode–electrolyte interface is governed by the electrode's chemical potential (µa) and the electrolyte's lowest unoccupied molecular orbital (LUMO) energy level (Figure 2a) [29]. Owing to the exceptionally low chemical potential of metallic lithium (−3.04 V vs the standard hydrogen electrode), achieving chemical compatibility with most solid electrolyte systems is inherently challenging. As a result, sulfide electrolytes undergo spontaneous redox reactions upon contact with Li metal, producing various decomposition species at the Li/SE interface (Figure 2b,d) [37]. Electrochemical impedance spectroscopy further reveals that this interfacial reaction is accompanied by a rapid and continuous increase in interfacial resistance. As shown in Figure 2c, time‐dependent Nyquist plots of symmetric Li/LPS/Li cells exhibit a progressive increase in overall impedance with contact time, which can be assigned to the formation and thickening of a resistive interphase at the Li/sulfide SE interface [25]. These reactions typically yield chemically heterogeneous interphases that can be either kinetically stabilized or continue to evolve depending on the electrolyte chemistry and cycling conditions, driving chemo‐mechanical degradation, electrolyte consumption, and rising interfacial impedance [38]. For instance, LGPS, LPS, and argyrodite‐type LPS*X* readily break down to Li_2_S, Li_3_P, S, GeS_2_, and LiCl upon contact with Li metal [17]. In situ spectroscopy and imaging studies further demonstrate that interphase formation induces void formation and contact loss during Li stripping, leading to current localization and lithium filament growth upon subsequent plating (Figure 2d,e), ultimately limiting the critical current density (CCD) and causing premature cell failure [33, 36]. With prolonged cycling, continuous accumulation of such interfacial byproducts, which exhibit lower Li‐ion conductivity and higher electronic conductivity than the pristine electrolyte, impede Li‐ion transport, increase interfacial resistance, accelerate dendrite propagation, and ultimately lead to severe performance degradation [7, 25, 39]. Therefore, the stable long‐term operation of sulfide‐based ASSLBs fundamentally relies on engineering robust interphases or artificial interlayers that decouple chemical instability from mechanical failure and enable practical operation beyond the intrinsic thermodynamic stability limits of sulfide electrolytes [35].

**FIGURE 3:**
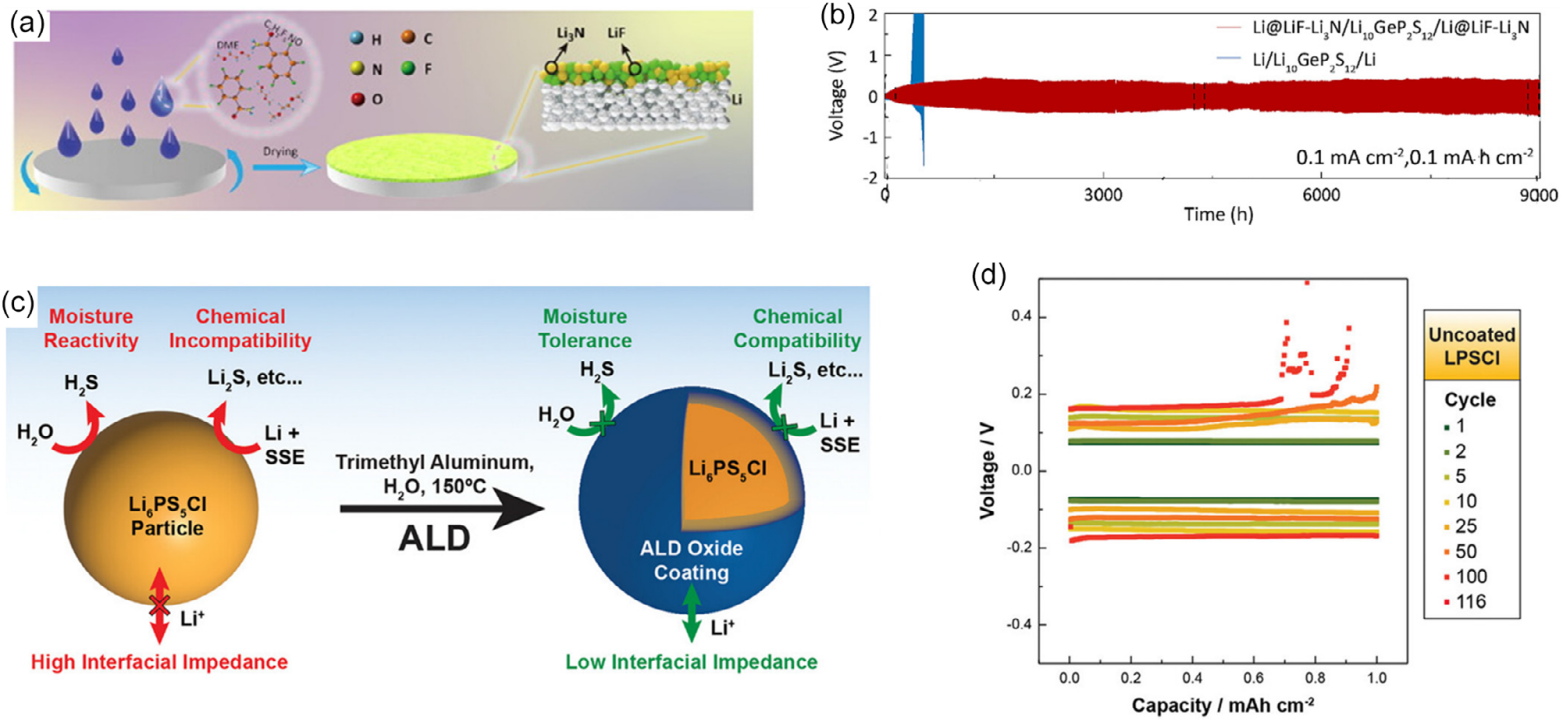
(a) Illustration of uniform LiF‐Li_3_N type interphase formation facilitated by interfacial modification (b) Long‐term symmetric‐cell voltage profiles demonstrating stable Li plating/stripping due to LiF‐Li_3_N interlayer, indicating suppressed parasitic reactions and enhanced interfacial stability [43] Stabilization of the Li metal/sulfide SE interface via ultrathin ALD ceramic coatings [45] (c) Schematic illustration showing that conformal ALD oxide coatings on LPSCl particles suppress moisture sensitivity and chemical reactivity while reducing interfacial impedance with Li metal. (d) Improved cycling stability of Li/LPSCl/Li symmetric cells enabled by ALD‐coated LPSCl compared with uncoated electrolyte.

The introduction of artificial interfacial layers applied either in situ or ex situ has become one of the most widely adopted strategies to address the instability between the Li metal and sulfide SE interface [40]. These approaches aim to regulate interfacial chemistry, suppress parasitic reactions, homogenize Li^+^ flux, and mitigate chemo‐mechanical degradation. Such interface engineering strategies have been extensively explored in sulfide‐based ASSLBs and are widely recognized as essential for maintaining low‐impedance and uniform Li plating/stripping behavior [10, 38]. A range of inorganic interlayers—including oxides, polyanionic oxides, nitrides, and metallic coatings—as well as polymeric interlayers have been employed to stabilize the Li/SE interface. Inorganic materials such as oxides and nitrides offer high mechanical robustness and chemical stability, enabling physical suppression of lithium filament penetration and interfacial degradation [41, 42]. In particular, electronically insulating yet Li‐ion‐conducting inorganic phases are effective in decoupling Li^+^ transport from electron transfer at the interface, thereby suppressing continuous electrolyte reduction [7, 13]. Inorganic Li‐ion‐conducting layers such as Li_3_N, LiF, and Li_2_O suppress sulfide decomposition by forming electronically insulating yet ionically conductive barriers at the Li/SE interface [43, 44]. Their properties can also be tailored—such as through doping—to enhance Li^+^ conductivity or improve interfacial compatibility. For example, Yao et al. [43] demonstrated an in situ formed LiF‐Li_3_N composite layer generated by reacting pentafluorobenzamide with Li metal (Figure 3a,b). This LiF‐Li_3_N interphase, characterized by high interfacial energy and strong adhesion, effectively suppressed parasitic reactions and dendrite formation, leading to uniform Li deposition. With this protective layer, the CCD of LGPS and LPSCl increased to 3.25 and 1.25 mA cm^−2^, nearly three‐ and two‐fold higher than symmetric cells lacking the coating. And the Li@LiF‐Li_3_N/LGPS/Li@LiF‐Li_3_N symmetrical cells with coating cycled stably for 9000 h at 0.1 mA cm^−2^ under 0.1 mAh cm^−2^. This work exemplifies how rationally designed inorganic interphases can simultaneously enhance interfacial stability and enable higher current density operation in sulfide‐based ASSLBs. Ultrathin ceramic films deposited by atomic layer deposition (ALD), including Al_2_O_3_, LiAlO_2_, and LiNbO_3_, have been shown to homogenize current distribution and reduce interfacial stress, dramatically increasing long‐term stability of Li/Li symmetrical cells and CCD values [42, 45]. For example, conformal ALD alumina coatings applied to LPSCl particles effectively suppressed chemical reactivity with Li metal and reduced interfacial impedance without inducing significant structural degradation of the sulfide electrolyte (Figure 3c,d) [45]. As a result, Li/LPSCl/Li cells exhibited substantially improved room‐temperature cycling stability, sustaining ≥ 150 cycles at high areal capacity (1 mAh cm^−2^ per cycle) and current density (0.5 mA cm^−2^). These improvements were attributed to modified intergranular chemistry and enhanced Li metal wetting at the electrolyte interface, underscoring the effectiveness of ALD‐derived ceramic coatings for stabilizing sulfide electrolytes under aggressive operating conditions.

While surface‐derived ceramic coatings improve interfacial chemistry and mechanical robustness, complementary strategies targeting the anode architecture itself have also emerged to regulate lithium deposition behavior more directly. Beyond surface‐applied interlayers, anode‐architecture‐driven strategies have emerged as an effective approach to stabilize the Li/sulfide solid electrolyte interface under practical operating conditions. Silver–carbon (Ag–C) composite anodes provide lithiophilic nucleation sites that homogenize Li plating and stripping, suppress void formation, and mitigate current localization at the interface [46]. In a recent study, Lee et al. demonstrated high‐energy, long‐cycling ASSLBs enabled by Ag–C composite anodes, achieving stable cycling at high areal capacities and extended cycle life in sulfide‐based solid‐state cells [16]. This work highlights that regulating Li deposition behavior through anode architecture, in addition to interfacial coatings, is critical for realizing durable sulfide‐based ASSLBs.

Recent operando studies of thin metallic interlayers on LPSCl‐based systems have systematically compared metallic interlayers including Ag, Mg, In, and Bi, revealing how alloying thermodynamics, phase transformation behavior, and lithium diffusivity collectively govern interfacial stability [38]. Operando scanning electron microscopy (SEM) demonstrated that Ag and Mg, which form solid‐solution‐type Li alloys, exhibit more homogeneous lithiation and maintain structural integrity during cycling, whereas hard intermetallic‐forming materials like Bi can fracture due to large volume expansion (∼60%) upon lithiation. Indium, despite forming intermetallic phases, remains mechanically stable owing to its intrinsic softness and relatively smaller volumetric mismatch. All metallic interlayers reduced lithium nucleation overpotential and promoted more globular and uniform plating compared to bare interfaces, although electronically conductive interlayers also increased the electrochemically active area and accelerated SEI formation. These findings suggest that effective metallic interlayer design should balance alloying thermodynamics, phase‐transformation homogeneity, lithium diffusivity across composition ranges, mechanical robustness, and SEI compatibility. Such mechanistic insight provides practical design criteria for selecting lithiophilic metallic interlayers to stabilize Li/sulfide SE interfaces and inform rational interlayer selection under realistic operating conditions.

In addition to purely metallic interlayers, hybrid organic–inorganic protective designs have been explored to combine mechanical compliance with chemical stability at the Li/sulfide interface. Composite protective layers integrate the benefits of both polymeric and inorganic components, typically combining a polymer matrix with inorganic fillers to achieve balanced mechanical robustness, chemical stability, and Li^+^ transport properties. When properly engineered, such hybrid layers can suppress dendrite growth, stabilize the interface, and facilitate efficient ion transport. Wu et al. [47] developed a superhydrophobic Li‐conducting protective layer for sulfide SE membranes by spray‐coating fluorinated polysiloxane‐modified Li_1.4_Al_0.4_Ti_1.6_(PO_4_)_3_ (F‐POS@LATP) nanoparticles. In this design, LATP nanoparticles provide continuous Li^+^ transport channels and microscale roughness, while the fluorinated polysiloxane coating generates a superhydrophobic, low‐surface‐energy network that binds the particles and protects the sulfide electrolyte from moisture attack. As a result, the otherwise moisture‐sensitive sulfide electrolyte membrane (LPSCl) could withstand extreme exposure conditions, including direct water jetting under high humidity (∼70% RH), without structural or electrochemical degradation. Importantly, despite acting as a superhydrophobic barrier, the F‐POS@LATP layer remains Li^+^‐conductive and introduces only a moderate increase in total resistance. Increasing the coating thickness led to a gradual rise in resistance from 5.32 Ω for the bare membrane to 9.95–14.2 Ω, attributable to the comparatively lower ionic conductivity of the coating layer, yet without compromising overall cell performance. Full ASSBS configured as Li_4_Ti_5_O_12_/ F‐POS@LATP/LPSCl/F‐POS@LATP membranes/LiNbO_3_‐coated LiCoO_2_ delivered a reversible capacity of 147.3 mAh g^−1^, comparable to cells using pristine sulfide membranes, and maintained stable cycling up to 80 cycles, as well as unchanged rate performance even after extreme environmental exposure. This work, therefore, represents a membrane‐level stabilization strategy specifically for sulfide solid electrolytes, enabling large‐area sulfide SE membranes to be transported, stored, and processed in air without inert‐atmosphere protection, addressing a critical commercialization bottleneck of sulfide‐based ASSLBs.

Among various interface‐engineering strategies, polymer‐based interlayers have gained considerable attention because of their mechanical flexibility, ease of processing, and ability to maintain intimate contact at the Li/sulfide electrolyte interface [48, 49]. These soft and compliant layers help accommodate volume changes during Li plating/stripping, reducing interfacial contact loss, particularly under low stack pressure. In addition, many polymers exhibit intrinsic Li^+^ conductivity, enabling smoother ion transport across the interface. Recent studies suggest that polymer interlayers can actively modulate interfacial chemistry. For example, investigations on the interphase formed between argyrodite‐type LPSCl and PEO_10_:LiTFSI reveal that the polymer modifies the decomposition pathway of the sulfide electrolyte, leading to the formation of a more ionically conductive and chemically stable interphase layer rather than extensive formation of insulating sulfide byproducts [50]. This engineered interphase reduces impedance buildup and enhances Li^+^ transport, demonstrating that polymer interlayers can simultaneously improve mechanical contact and tailor interfacial chemistry. However, a deeper understanding of the chemical interplay between solid polymer electrolytes and LPSCl remains necessary to assess the time‐ and temperature‐dependent evolution of solid polymer electrolyte interphases.

Beyond artificial coatings, self‐formed interphases generated by controlled electrolyte decomposition have also been shown to kinetically stabilize the Li|sulfide interface. Such reaction‐derived interphases, although thermodynamically unstable, may kinetically suppress further electrolyte decomposition by forming electronically blocking yet ionically permeable layers that regulate Li deposition behavior. Despite these advances, key challenges remain, including optimizing interlayer thickness, maintaining low interfacial impedance under practical current densities, and ensuring long‐term mechanical integrity under repeated stripping and plating. A deeper understanding of the coupled electrochemical–mechanical evolution of Li metal interphases, particularly under extended cycling and elevated temperature, will be essential for translating these interlayer concepts to practical ASSLB architectures. Collectively, these strategies demonstrate that controlling interfacial chemistry, Li deposition behavior, and mechanical stability is essential for achieving stable Li metal operation with sulfide‐based electrolytes.

## 3 Engineering of Cathode/Sulfide SE Interfaces

Sulfide SEs, when paired with high‐capacity layered oxide cathodes such as LiNi_1–*x*–*y*_Mn_*x*_Co_*y*_O_2_ acronymic NMC or lithium nickel–cobalt–aluminum oxide (LiNi_1–*x*–*y*_Co_*x*_Al_*y*_O_2_, in short NCA) and their derivatives, face severe interfacial degradation, which remains a major obstacle to practical implementation [51]. The electrochemical instability of sulfide SEs against oxide‐based cathodes leads to the formation of irreversible, resistive passivation layers at the cathode/SE interface, which impede Li‐ion transport and rapidly increase interfacial resistance [51, 52]. Additionally, chemo‐mechanical fractures arising from volume and stress mismatches cause physical contact loss at the interface, further hindering Li‐ion migration from the earliest cycles [27]. During cycling, most intercalation‐type cathodes undergo ∼5% volume fluctuations, which can induce cracking within secondary particles. The newly exposed surfaces often lose intimate contact with the sulfide electrolyte, leading to increased interfacial resistance and a reduction in reversible capacity [53]. As a result, the electrochemical performance of sulfide‐based ASSLBs is often dominated by cathode–electrolyte interfacial resistance rather than bulk transport properties.

**FIGURE 4:**
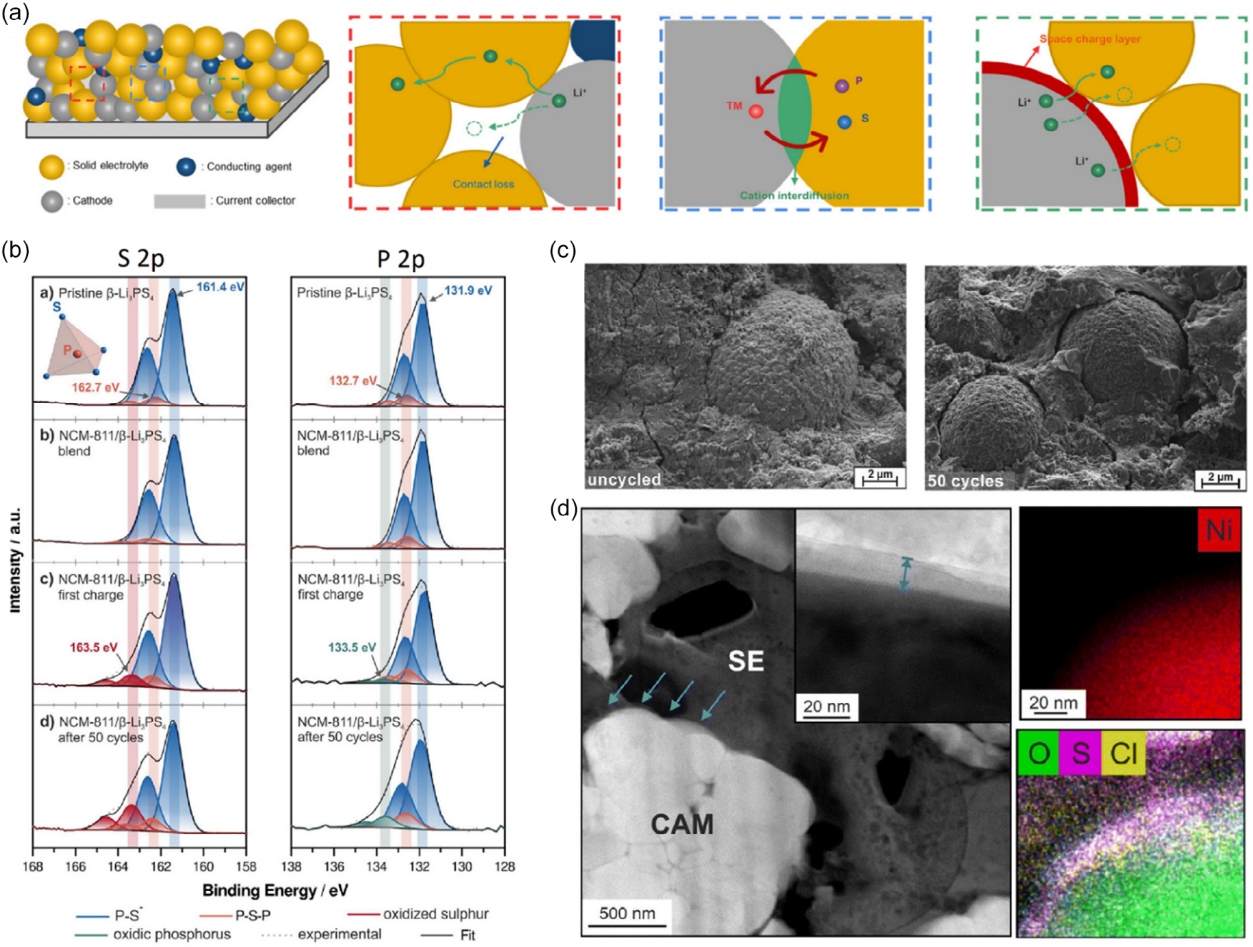
Major degradation mechanisms at the cathode–sulfide SE interface in ASSLBs. (a) Schematic illustration summarizing interfacial contact loss, cations interdiffusion, and space‐charge layer formation at layered oxide cathode/sulfide solid electrolyte interfaces. (b) High‐resolution S 2p and P 2p XPS spectra of NMC811, β‐Li_3_PS_4_ composites showing progressive oxidative decomposition of the thiophosphate electrolyte upon first charge and prolonged cycling, evidenced by the emergence and growth of oxidized sulfur and phosphorus species. (c) SEM images revealing chemo‐mechanical degradation of the composite cathode, including cathode particle contraction and loss of intimate contact with the sulfide electrolyte after cycling [51]. (d) Cross‐sectional cryo‐STEM and EDS analysis of the cycled cathode, including a HAADF overview image showing both the cathode active material (CAM) and solid electrolyte (SE), with the interfacial region of interest indicated by arrows and corresponding elemental maps (Ni, O, S, and Cl), revealing transition‐metal interdiffusion and interfacial chemical heterogeneity [54].

Despite extensive study, the mechanisms governing interphase formation and degradation remain incompletely understood, posing a critical challenge for the development of high‐energy sulfide‐based ASSLBs. A clear understanding of the interfacial phenomena that drive this degradation is therefore crucial for advancing sulfide‐based ASSB technologies. As illustrated in Figure 4a, the major challenges at the cathode/sulfide SE interface include interfacial contact loss induced by cathode volume changes during cycling, cation interdiffusion across the interface, and the development of space‐charge layers (SCLs) that promote oxidative decomposition of sulfide electrolytes at high voltages [55, 56].

Interfacial degradation between high‐voltage layered oxide cathodes and sulfide SEs becomes particularly pronounced when the cathode is charged above ∼3.8 V vs. Li^+^/Li. In NMC811, β‐ Li_3_PS_4_ systems, this high‐voltage regime drives the oxidative breakdown of the thiophosphate electrolyte, leading to the formation of an interfacial cathode–electrolyte interphase (CEI). XPS spectra represented in Figure 4b clearly illustrate this process: while the pristine β‐Li_3_PS_4_ and the uncycled NMC811/SE blend show only the expected P—S and P—S—P bonding environments, new high‐binding‐energy features emerge after the first charge. These additional peaks correspond to oxidized sulfur species (e.g., S–S or polysulfide‐like environments) and oxidized phosphorus species, indicating the onset of electrolyte decomposition at the charged interface. Upon prolonged cycling, these oxidized components intensify and broaden, demonstrating progressive CEI thickening and increasing chemical complexity [51]. SEM observations (Figure 4c) further demonstrate that cathode particle contraction during delithiation disrupts physical contact with the sulfide electrolyte, contributing to impedance growth and capacity decay. Together, these observations highlight that both interfacial chemical instability and mechanically driven contact loss critically undermine the performance of sulfide‐based ASSLBs employing high‐Ni layered cathodes [51]. Maintaining stable physical contact between cathode active materials and sulfide electrolytes, therefore, remains a fundamental challenge, owing to the rigid, brittle nature of sulfide electrolytes and the complex chemo‐mechanical behavior of layered oxide cathodes [57, 58].

Cation interdiffusion constitutes another key interfacial degradation pathway at sulfide electrolyte–layered oxide cathode interfaces [59]. Early work by Sakuda et al. revealed that, even during the initial charge, Co migrates from LiCoO_2_ (LCO) into sulfide electrolytes such as 80Li_2_S·20P_2_S_5_, forming a chemically intermixed region as evidenced by the high‐angle annular dark‐field (HAADF)‐scanning transmission electron microscopy (STEM)‐based energy dispervise x‐ray spectroscopy (EDS) results [26]. Subsequent computational studies extended this understanding, showing that sulfide glasses and crystalline sulfides, including LGPS, exhibit strong thermodynamic driving forces for transition‐metal diffusion when interfaced with LCO and related high‐voltage cathodes [60, 61]. More recent cryogenic TEM studies provided direct, nanoscale visualization of transition‐metal interdiffusion from Ni‐rich layered oxides into thiophosphate electrolytes during realistic cycling conditions (Figure 4d) [54]. These studies showed that Ni/Co dissolution and migration lead to the formation of mixed‐metal sulfide phases at the interface, which act as electronically conductive poison layers. These phases accelerate electrolyte decomposition, destabilize the cathode/electrolyte interface, and drive rapid interfacial resistance growth, consistent with the experimentally observed impedance rise in sulfide‐based ASSLBs [59]. Notably, such electrolyte poisoning occurs within the first few cycles, indicating that cation interdiffusion is an inherent interfacial instability rather than a long‐term degradation process.

In sulfide electrolyte–oxide cathode systems, the formation of a SCL at the interface presents another significant challenge [62]. At the cathode/sulfide SE interface, differences in chemical and electrostatic potentials drive lithium ions away from the interface, creating a Li‐depleted SCL [63, 64]. This Li‐deficient region impedes Li^+^ transport, increasing interfacial resistance and polarization [63]. With repeated cycling, SCL thickening further degrades rate capability and cycling stability [55]. Consequently, mitigating SCL formation is essential for achieving stable and efficient sulfide‐based ASSLBs. Beyond electrostatic effects associated with SCL formation, chemical instability at the cathode/sulfide SE interface further exacerbates interfacial degradation. Oxygen release from layered oxide cathodes during early cycles leads to severe interfacial degradation in sulfide‐based solid‐state batteries. When oxygen is released at regions where the thiophosphate‐based sulfide SEs (Li_2_S–P_2_S_5_) directly contact the cathode, it triggers decomposition of the electrolyte into PO_*x*_ and MS_*x*_ species and simultaneously forms antisite defect layers on the cathode surface. These irreversible passivation layers disrupt Li‐ion transport and result in capacity fading and rising interfacial resistance [65].

**FIGURE 5:**
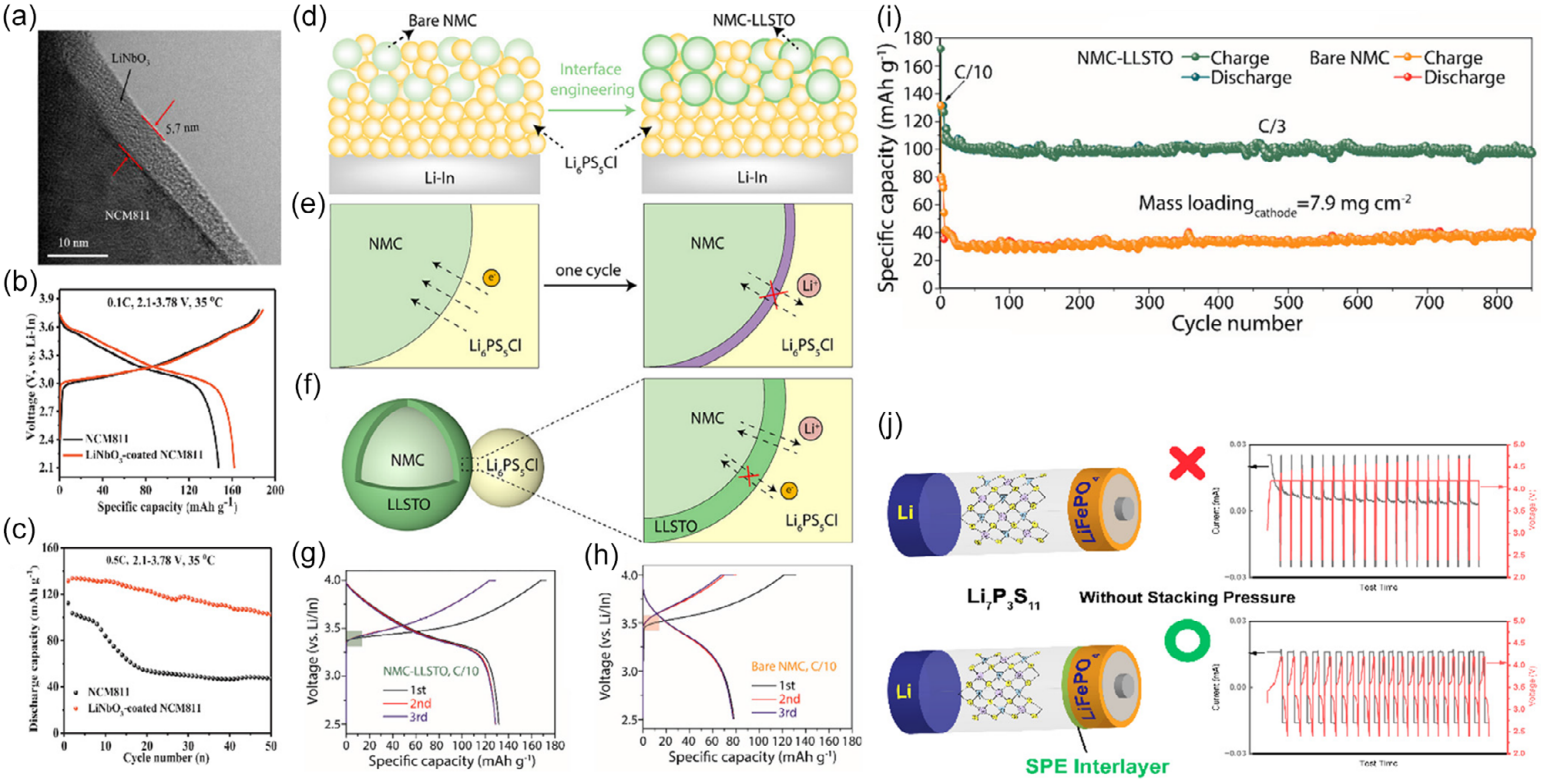
Interface engineering strategies for stabilizing oxide cathodes and sulfide SE‐based ASSLBs. (a) Cross‐sectional TEM image showing a conformal LiNbO_3_ coating on NMC811 particles. (b, c) Comparison of voltage profiles and cycling performance of bare NMC811 and LiNbO_3_‐coated NMC811, demonstrating suppressed interfacial degradation at elevated temperature. (d–f) Schematic illustration of interface engineering using an LLSTO interlayer at the NMC/LPSCl interface, highlighting the formation of a stabilized interphase after initial cycling. (g,h) Charge–discharge voltage profiles of LLSTO‐coated NMC versus bare NMC, showing improved reversibility and reduced polarization. (i) Long‐term cycling performance of bare NMC and NMC–LLSTO composite cathodes evidencing significantly enhanced cell performance enabled by the LLSTO interlayer. (j) Comparison of Li_7_P_3_S_11_‐based ASSLB cycled without external stack pressure, illustrating that the introduction of a solid polymer electrolyte (SPE) interlayer improves interfacial contact and enables stable cycling under pressure‐free conditions.

To address the fundamental interfacial instability between sulfide electrolytes and cathode materials, several strategic directions have emerged, including (1) employing surface coatings and buffer layers on cathode active materials, (2) tailoring the composition and chemistry of sulfide electrolytes, and (3) designing next‐generation cathode architectures [59]. A representative approach to stabilizing the cathode–sulfide electrolyte interface is the application of thin inorganic surface coatings. For example, ZrO_2_‐coated LiNi_1/3_Mn_1/3_Co_1/3_O_2_ (NMC111), prepared through a sol–gel process assisted by ultrasonic irradiation, has been shown to markedly improve the performance of ASSLBs using amorphous Li_3_PS_4_ as the SE. The ZrO_2_ layer effectively suppressed interfacial resistance growth during cycling, enabling more stable charge–discharge behavior. As a result, cells incorporating the ZrO_2_‐coated NMC delivered an initial discharge capacity of ∼ 115 mAh g^−1^ and retained their capacity over 50 cycles at 0.1 mA cm^−2^ at room temperature, demonstrating the value of interface‐protective coatings in enhancing long‐term electrochemical stability [66]. Applying a uniform and conformal LiNbO_3_ coating on the cathode surface effectively suppresses oxygen release at the cathode‐electrolyte interface. Where the LiNbO_3_ layer is well formed, oxygen evolution is significantly reduced, preventing the formation of PO*_x_*/MS*_x_* decomposition products and inhibiting antisite defect generation on NMC111. This coating maintains a stable interface, preserves Li‐ion conduction pathways, and mitigates long‐term capacity fading. The findings emphasize that coating integrity and adhesion are critical: only a continuous, stable LiNbO_3_ interlayer can prevent oxygen‐induced degradation throughout extended cycling [65]. As shown in Figure 5a–c, a thin layer of LiNbO_3_ coating on NMC811 markedly improved electrochemical performance compared with bare NMC811 in LGPS‐based ASSLBs, including higher initial discharge capacity, enhanced Coulombic efficiency, superior rate capability, and significantly improved cycling stability [67]. The significant improvement in battery performance is achieved due to the conformal LiNbO_3_ buffer layer coating that effectively stabilizes the cathode/sulfide SE interface by suppressing interfacial degradation and facilitating Li^+^ transport under high current densities. Li_2_CO_3_/LiNbO_3_ coatings on layered oxide cathodes have been shown to effectively suppress interfacial degradation when paired with thiophosphate solid electrolytes. The coating reduces the formation of oxygenated phosphorus and sulfur species—such as phosphates, sulfates, and sulfites—that typically arise from oxidative decomposition of the electrolyte at high voltages. Electrochemical measurements confirm that this protective layer mitigates capacity fading and significantly improves long‐term cycling performance in sulfide‐based ASSBs. Postmortem XPS and time of flight secondary ion mass spectroscopy (ToF‐SIMS) analyses further reveal that the coating limits the successive buildup of decomposition products within the interfacial region. However, because the particulate Li_2_CO_3_/LiNbO_3_ coating does not fully cover all cathode surface sites and is not perfectly stable against the electrolyte, gradual degradation still occurs over extended cycling. These findings highlight both the effectiveness and the remaining limitations of oxide‐based coatings in stabilizing the cathode–sulfide electrolyte interface [68]. As illustrated in Figure 5d–i, introducing a thin amorphous Li_0.35_La_0.5_Sr_0.05_TiO_3_ (LLSTO) interlayer at the NMC/LPSCl interface fundamentally alters the interfacial reaction pathway, effectively suppressing sulfide electrolyte decomposition at high voltage and stabilizing Li^+^ transport across the cathode–electrolyte interface [69]. This interlayer‐enabled interface engineering resulted in markedly reduced polarization, improved rate capability, and exceptional long‐term cycling stability, enabling high areal‐loading ASSLBs to operate stably over hundreds of cycles at elevated voltages where bare NMC/sulfide interfaces rapidly fail.

Apart from the aforementioned inorganic surface coatings, the insertion of a thin solid polymer electrolyte (SPE) interlayer at the cathode/sulfide SE interface has also emerged as an effective strategy for mitigating interfacial challenges. Owing to their mechanical compliance and good interfacial wettability, SPE interlayers can accommodate chemo‐mechanical mismatches and maintain intimate contact between the cathode active material and the sulfide electrolyte. For example, a PVDF–HFP–based SPE interlayer has been introduced at the LiFePO_4_ (LFP)/sulfide SE interface (Figure 5j). The inclusion of the SPE interlayer at cathode/sulfide SE interface enabled stable cycling of ASSLBs without applying any protective coating on the cathode active materials and under zero external stack pressure, highlighting the ability of polymer interlayers to decouple mechanical stabilization from chemical passivation at the cathode/sulfide SE interface [70].

Collectively, these studies demonstrate that thin, conformal inorganic coatings represent an effective and widely adopted strategy for mitigating interfacial degradation at the cathode–sulfide electrolyte interface. Oxide‐based interlayers such as ZrO_2_ and LiNbO_3_ can suppress electrolyte oxidation, limit oxygen release from layered oxide cathodes, and reduce the formation of resistive interphases, thereby improving cycling stability in sulfide‐based ASSBs. However, their effectiveness strongly depends on coating uniformity, interfacial adhesion, and chemical stability under prolonged high‐voltage operation. Incomplete surface coverage or gradual chemical degradation can still lead to interfacial failure over extended cycling. These limitations underscore the need for next‐generation cathode interlayers that combine chemical robustness with mechanical compliance and scalable processing, motivating continued exploration of hybrid, self‐forming, and adaptive interlayer architectures.

## 4 Engineering of Li Metal Anode/Halide SE Interfaces

Halide SEs have become the promising SEs for ASSBs alongside sulfide and oxide SEs after Asano et al. reported lithium halide SEs, Li_3_YCl_6_ (LYC) and Li_3_YBr_6_ (LYB), to exhibit high lithium‐ion conductivity, high deformability, and high chemical and electrochemical stability in 2018 [19]. They reported excellent performance of ASSBs in terms of Coulombic efficiency and cycling performance using In‐Li alloy as anode and 4 V class LCO cathode active materials without any additional protective coating on cathode active materials (CAMs). The high initial Coulombic efficiencies of 94.8% for the LYC cell and 94.2% for the LYB cell were taken as a clear indication that the halide SEs are highly stable against 4 V class cathode active materials. After this, many HSEs, typically represented by Li_3_MX_6_ (*M* ^3+^ = In, Y, Sc; *X* = Cl, Br, I), and their doped derivatives have been reported, which uniquely integrate the advantages of both sulfide and oxide electrolytes, offering high room‐temperature ionic conductivities (0.1–10 mS cm^−1^), broad oxidative stability up to ∼4.5–5.0 V comparable to ceramic oxides, and exceptional mechanical deformability and processability similar to sulfide‐based systems [21, 71, 72]. This combination of excellent electrochemical robustness and favorable manufacturability positions HSEs among the most promising candidates for enabling high‐energy‐density ASSLBs [24, 73, 74, 75].

**FIGURE 6:**
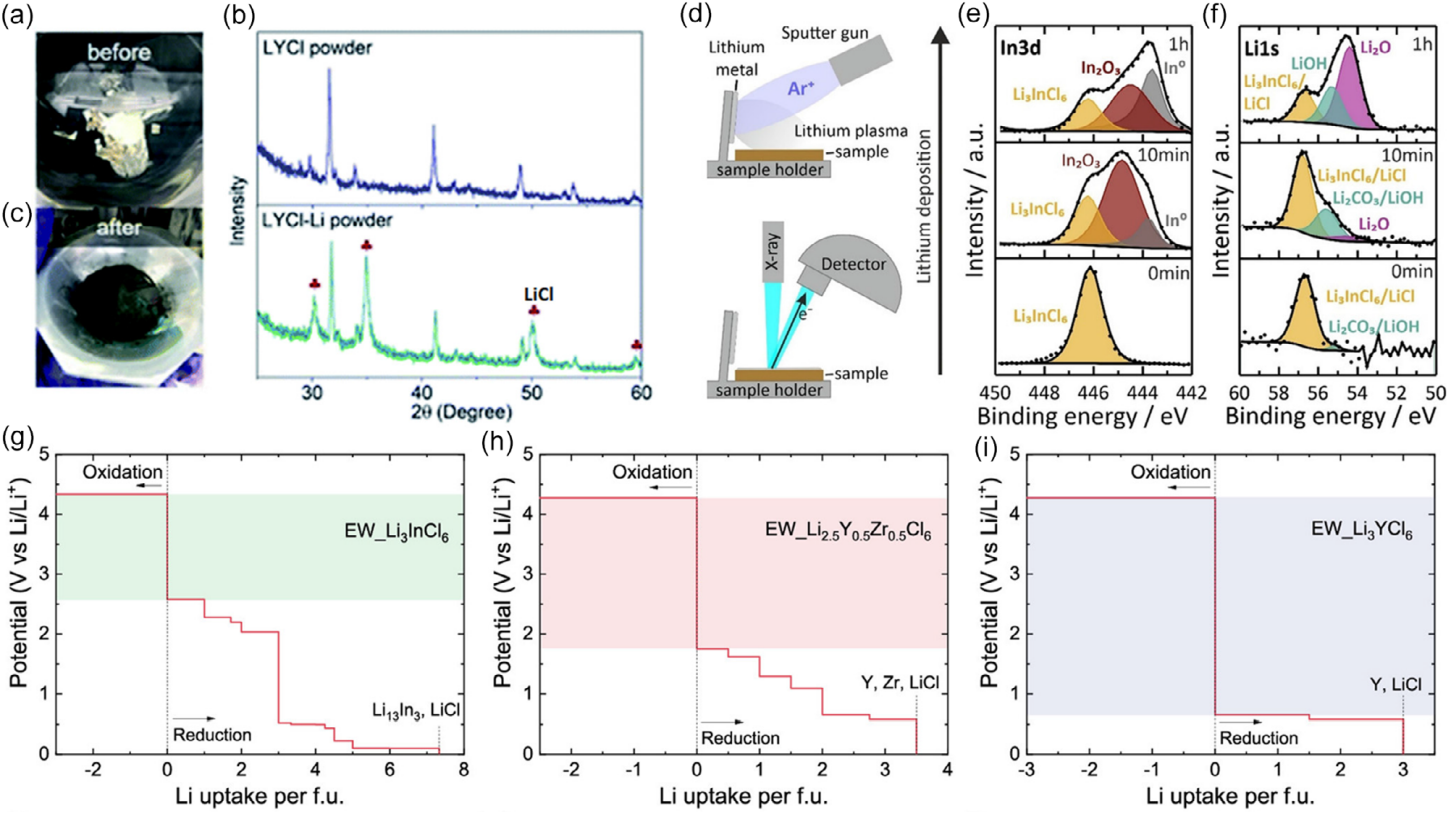
Interfacial instability between Li metal and halide SEs. (a–c) [76] Visual and x‐ray diffraction [77] evidence of chemical degradation of LIC (or LYC) after contact with Li metal, showing the formation of LiCl and other reaction products. (d) Schematic of operando Li deposition during XPS measurements. (e,f) Operando XPS In‐3d and Li‐1s spectra reveal rapid reduction of the halide electrolyte, forming metallic In^0^, LiCl, and Li–O species (Li_2_O/LiOH/Li_2_CO_3_). The progressive loss of pristine halide signals and growth of decomposition products demonstrate the formation of a nonpassivating, mixed‐conducting interphase at the Li/halide interface [24]. Potential profiles for oxidation and reduction reactions for halide SEs. (g) Li_3_InCl_6_. (h) Li_2.5_Y_0.5_Zr_0.5_Cl_6_. (i) Li_3_YCl_6_, including the electrochemical stability window (colored region) and phase equilibria with lithium metal.

Yet, despite these favorable bulk characteristics, achieving a stable interface between HSEs and Li‐metal remains a critical bottleneck for their commercialization. Recent experimental and computational studies have revealed that HSEs undergo interfacial decomposition when in direct contact with Li metal, leading to a continuously growing interphase resistance and failure under high current densities. Janek et al. demonstrated experimentally, using in situ XPS combined with impedance spectroscopy, that the interface between Li_3_InCl_6_(LIC) or LYC and lithium metal is thermodynamically unstable and results in a continuously growing interphase resistance due to the presence of LiCl and metallic phase [24]. Operando XPS results, as shown in Figure 6d–f, show that LIC undergoes rapid reduction when contacted by Li metal. In the In‐3d region, the pristine In^3+^ signal quickly gives way to metallic In^0^ and In_2_O_3_ within minutes, indicating reduction by Li^0^ and subsequent oxidation of In^0^ by trace oxygen. After extended Li deposition, the LIC peak is strongly diminished, demonstrating progressive consumption of the electrolyte and burial beneath a growing interphase. Correspondingly, the Li‐1s spectra evolve from LIC/LiCl features to dominant Li_2_O, LiOH, and Li_2_CO_3_ signals, reflecting reactions between Li metal, decomposition products, and residual impurities. Overall, the XPS trends confirmed that LIC does not form a stable passivating interphase at the Li interface; instead, Li continuously reduces the halide framework, producing a thick, mixed‐conducting layer composed of In^0^, LiCl, and Li‐O species. Their in situ deposition experiments proved that halide SEs, including both LIC and LYC, exhibit rapid decomposition at the lithium metal anode following the reaction Li_3_ *M*Cl_6_ + 3Li→6LiCl+*M* ^0^ (*M* = In, Y) and cannot be used as separator electrolyte materials in SSBs without interfacial protection.

A central challenge in this degradation process is the formation of mixed ionic–electronic conducting (MIEC) interphases. Unlike sulfides, which typically decompose to electronically insulating Li_2_S/Li_3_P passivation layers, halide electrolytes generate conductive metal nanoparticles (In, Y, or Sc) upon reduction. These metallic species initially nucleate at the immediate Li/halide interface and, with continued reduction, undergo growth and agglomeration. As the local volume fraction of metallic particles increases, interparticle contact and electron tunneling between adjacent nanoparticles can occur, eventually reaching a percolation threshold that establishes continuous electronic conduction pathways across the interphase. These metallic species create electron percolation pathways across the interface, promoting sustained electrolyte reduction, interphase thickening, increasing polarization, and progressive cell performance degradation under moderate to high current densities [24, 77]. The origin of this instability lies in the thermodynamic driving force for the reduction of metal cations (In^3+^, Y^3+^, Sc^3+^) and halide anions (Cl^‐^) when exposed to strongly reducing Li^0^. First‐principles thermodynamic analyses also show that HSEs are thermodynamically metastable against Li metal, where Li^0^ can reduce *M* ^3+^ to metallic phase while simultaneously forming LiCl and complex lithium–chloride phases (Figure 6g–i), generating a mixed‐conducting interphase that accelerates interfacial growth and causes high interface resistance and electrolyte/electrode depletion [24, 78, 79, 80]. Operando XPS and ToF‐SIMS studies similarly confirm the formation of In^0^, LiCl, Li_2_O, and, depending on trace oxygen/moisture content, oxychloride species that accumulate into a resistive but electronically percolating interphase, with spatially distributed metallic domains embedded within LiCl‐rich matrices, consistent with the formation of interconnected electron migration paths [24]. Understanding the chemistry and dynamics of the interface between halide and Li metal is necessary to design interlayers ensuring sufficient contact, efficient carrier transport, and dendrite suppression, which are key for a stable cell performance [81].

To stabilize the interface between Li metal anode and halide SEs, a thin sulfide SE layer is often inserted as a buffer to prevent direct contact between the two. Such an intermediate sulfide phase functions as a kinetically stable barrier that suppresses continuous reductive decomposition of halide electrolytes while maintaining fast Li^+^ transport across the interface. For example, previous studies have demonstrated that sulfide SEs such as LPSCl, LPS, and LGPS can serve as anodic interlayers, with LPSCl forming a stable, ionically conductive interface composed mainly of Li_2_S, Li_3_P, and P_2_S_5_ when in contact with Li metal, making it an attractive buffer layer [21, 71, 76]. Direct comparisons further underscore this effect: symmetric Li cells incorporating an LPSCl interlayer between Li metal and LYC exhibited stable Li plating/stripping overpotentials (∼100 mV for > 1000 h), whereas cells without the sulfide buffer showed rapid polarization buildup (∼1500 mV within 60 h), highlighting the critical role of sulfide interlayers in kinetically stabilizing Li|HSE interfaces [76]. Building upon these insights, diverse interlayer engineering strategies have been developed to stabilize Li/HSE interfaces by leveraging design principles established in sulfide‐based systems. One effective strategy involves introducing a lithiophilic, ionically conductive but electronically insulating interlayer that blocks electron transfer, directs Li‐ion flux, and buffers interfacial reactions.

**FIGURE 7:**
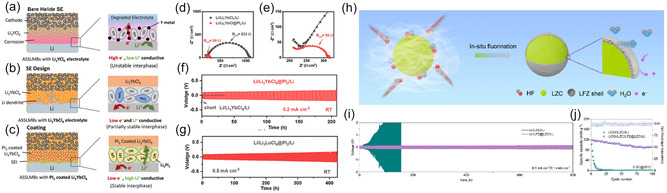
Interphase formation and stabilization at the Li/halide SE interface in all‐solid‐state lithium metal batteries (ASSLMBs). (a–c) Direct contact between Li and LYC leads to an electronically conductive, Li^+^‐poor interphase and poor cycling stability, whereas electrolyte design and PI_3_ coating induce the formation of a Li_3_P‐rich, electronically insulating, and Li^+^‐conductive interphase, significantly reducing interfacial resistance and improving symmetric‐cell performance [82]. (d) Electrochemical impedance spectroscopy profiles of as‐assembled Li/Li_3_YbCl_6_/Li cells and Li/Li_3_YbCl_6_@PI_3_/Li cells. (e) an enlarged view of (a). (f) Galvanostatic cycling of Li/Li_3_YbCl_6_/Li cells and Li/Li_3_YbCl_6_@PI_3_/Li cells at a constant current density of 0.2 mA cm^−2^ (g) Galvanostatic cycling of Li/Li_3_LuCl_6_@PI_3_/Li cells at a constant current density of 0.5 mA cm^−2^ (h) Fluorinated Li_2_ZrCl_6_ (LFZ@LZC) prepared via in situ gas–solid fluorination forms a LiF–ZrF_4_ shell that stabilizes the halide SE surface (i) Cycling performances of Li//LZC//Li and Li//LFZ@LZC//Li symmetric cells at 0.1 mA cm^−2^ and 0.1 mAh cm^−2^ (j) Cycling stability of the LCO//LZC//Li and LCO//LZC/LFZ@LZC//Li ASSLMBs at 0.2 C and 25°C [83].

Recently, a PI_3_ interlayer that converts into Li_6_PI_3_ upon contact with lithium has been shown to dramatically reduce the interfacial resistance of Li_3_YbCl_6_ and enable a high critical CCD to support stable cycling of Li‐symmetrical (Figure 7a–g) [82]. In another study, a silver interlayer was introduced by cold pressing, resulting in the formation of a Li–Ag alloy interface [84]. Through this buffer layer, direct contact between Li and LIC was prevented, thereby suppressing void formation and dendritic growth. Improved Li diffusion kinetics were achieved owing to the alloy phase, and a critical current density of 1.3 mA cm^−2^ was realized. Long‐term cycling stability was demonstrated, with symmetric Li–Ag/LIC/Li–Ag cells operating for 500 h at 0.1 mA cm^−2^. This approach has been presented as a simple and economical solution for stabilizing Li/halide SE interfaces.

In addition to metallic and halide‐derived interlayers, nitride‐based materials have also been employed to stabilize halide/Li interfaces. Commercial Li_3_N was transformed into α‐ and β‐phases by ball‐milling and annealing, respectively, with the β‐phase exhibiting both high room‐temperature ionic conductivity and chemical stability toward Li and Li_2_ZrCl_6_ [85]. When applied as an artificial interfacial layer, β‐ Li_3_N significantly reduced the impedance between Li_2_ZrCl_6_ and Li from 1929 to ∼400 Ω. At a current density of 0.1 mA cm^−2^, the overpotential of Li symmetric cells decreased from 250 to ∼50 mV and remained stable for at least 300 h. These results demonstrate that β‐ Li_3_N can serve as an effective interlayer material, providing both ionic transport and interfacial stability, thereby extending the design space for halide SEs in lithium‐metal batteries.

Among the various candidates explored so far, LiF‐constituted interphases have been shown to play a stabilizing role, as LiF is frequently formed in situ at Li, halide interfaces and can act as an electronically insulating, ionically permeable barrier that mitigates interfacial degradation [24]. A bilayer electrolyte composed of LIC and LPSCl was fabricated, and a LiF‐rich solid electrolyte interphase was preformed on Li metal. This dual design ensured stable interfacial contact and suppressed side reactions. When paired with an LCO cathode, the full cell retained over 85% of its initial capacity after 100 cycles at 0.2 C, demonstrating the synergistic role of the bilayer electrolyte and artificial LiF‐rich SEI in enabling long‐lasting performance [86]. A fluoride‐coated halide interlayer, exemplified by LiF–ZrF_4_@Li_2_ZrCl_6_ (LFZ@LZC), has recently been shown to significantly enhance the stability of Li/halide interfaces (Figure 7h) [83]. The fluoride‐rich shell acts as an ionically conductive but electronically insulating barrier, suppressing continuous reduction of the halide SE and lowering electronic conductivity by approximately one order of magnitude. As an interlayer, LFZ@LZC enables long‐term symmetric‐cell cycling (>800 h) while also improving full‐cell cycling stability even after air exposure (Figure 7i,j). These results highlight fluoride‐based coatings as a promising strategy for stabilizing HSE interfaces under demanding operating conditions.

Notably, recent studies indicate that interfacial decomposition of halide SEs does not universally lead to catastrophic failure but can, in specific chemistries, result in a kinetically stable and electrochemically functional interphase. In the mixed‐halide system Li_3_YCl_4_Br_2_, reaction with metallic Li induces partial reduction of the halide framework, forming LiCl, LiBr, and metallic Y^0^ [81]. During repeated cycling, these reduction products self‐organize into a structured solid electrolyte interphase comprising a Y‐rich electronically conductive network embedded within ionically conductive LiCl/LiBr domains. This chemically heterogeneous yet mechanically robust interphase exhibits mixed ionic–electronic conductivity and has been shown to sustain unexpectedly stable Li cycling, in stark contrast to the continuously growing, resistive interphases typically observed for single‐halide SEs such as LIC or LYC. Importantly, this behavior represents a chemistry‐specific stabilization mechanism, rather than a general feature of halide SEs, and underscores that controlled interfacial reactions when properly engineered may be harnessed to improve Li/halide compatibility rather than being universally detrimental.

In summary, while halide SEs offer compelling advantages for high‐voltage and high‐energy all‐solid‐state batteries, their interfacial instability against Li metal remains one of the most crucial obstacles to commercial adoption. Understanding the interplay between chemical reduction, interphase evolution, electron percolation pathways, and mechanical contact degradation is essential for designing robust interfaces. The most promising path forward involves integrating chemically stable interlayers, lithiophilic alloy buffers, and compositional tuning of the halide lattice into a unified interfacial‐engineering strategy. Continued development of operando characterization tools and multiscale computational methods will be vital to clarify the degradation mechanisms and rationally design next‐generation halide SEs capable of supporting dendrite‐free Li cycling at high rates and long lifetimes, ultimately enabling safe, energy‐dense solid‐state lithium‐metal batteries.

## 5 Engineering of Cathode/Halide SE Interfaces

Halide‐based SEs exhibit inherently strong compatibility with high‐voltage cathodes owing to the high oxidation potentials of halogen anions, which suppress electrolyte oxidation and minimize interfacial impedance. Early work demonstrated that chloride SEs such as LYC can operate directly with layered oxides like LCO without surface coatings, delivering significantly lower interfacial resistance and higher Coulombic efficiency compared to sulfide electrolytes. Subsequent studies have confirmed similar favorable compatibility with a wide range of layered Ni‐rich cathodes and high‐voltage spinel materials, enabling stable cycling up to ∼ 4.3 V in bulk‐type ASSLBs. These results highlight that the oxidative stability of HSEs is strongly influenced not only by halogen chemistry but also by the electronic structure of the central metal cation, which governs the onset of interfacial decomposition under deep delithiation. However, despite their generally robust high‐voltage performance, Halide SEs can still undergo interfacial redox reactions when paired with cathodes that trigger oxygen redox or aggressive surface reconstruction, especially when the potential is over 4.5 V, leading to the oxidative decomposition of HSEs and the formation of cathode‐electrolyte interface (CEI) comprising oxides, oxychlorides, or metal–halide reaction products that degrade stability at elevated cutoff voltages [79, 87]. It is important to note that this > 4.5 V regime typically exceeds the practical stable cycling window (∼4.3–4.4 V) demonstrated for most chloride‐based halide electrolytes in bulk‐type ASSLBs and therefore represents an overcharged or highly delithiated condition rather than standard operating voltage. Together, these findings demonstrate that while HSEs possess intrinsic advantages for cathode compatibility, their interfacial stability is ultimately dictated by the interplay between halogen chemistry, central‐metal oxidation behavior, and cathode surface reactions, requiring continued interface design to fully unlock their high‐voltage potential.

Strategies such as tuning the central metal composition, employing multicomponent halide chemistries, or partially fluorinating the electrolyte have proven effective in extending oxidation limits, suppressing interfacial degradation, and stabilizing high‐voltage operation. Moreover, the ability of HSEs – particularly fluoride‐containing compositions – to form passivating, ion‐conducting interphases at high potentials further underscores their promise as coatings or buffer layers for next‐generation high‐voltage cathodes. Such passivation typically occurs near the upper oxidation limit and may kinetically stabilize the interface, although it does not necessarily extend the intrinsic thermodynamic stability window beyond ∼ 4.5 V. Fluorine substitution has shown promising results to enhance the stability of HSEs, with proper composition tuning required for battery applications since excessively fluorinated HSE compounds may lack practical ionic conductivity. Fluorine substitutional doping can suppress the formation of oxychlorides, a parasitic product when paired with high‐voltage CAMs [88]. Fluorine‐substituted HSEs can also promote the formation of F‐containing passivating CEI, preserving the cycling stability of the battery [89]. Fluorine substitution of CAMs can stabilize their structure and lead to fewer cracks [90]. Doping the central metal element of HSE with a high‐valent element is also a possible approach, with a catholyte comprised of Li_2.6_In_0.8_Ta_0.2_Cl_6_ and NMC811 exhibiting 70% capacity retention after 950 cycles at 1.39 mA cm^−2^ with a voltage window of 3.0–4.4 V versus Li/Li^+^ [91]. The combination of fluorine and central metal element substitutional doping also improves moisture stability [72]. Collectively, these findings highlight the effectiveness of doping and coating strategies in stabilizing SE‐based interfaces under high‐voltage operation [74].

The crystalline size, orientation, morphologies, and defects are related to synthesis conditions and can affect the performance of both SE [92] and CAMs [93]. Microwave energy generated by magnetrons can induce molecular vibration, introducing heat from interior of the reaction media, and is seen as a promising approach to boost chemical reactions, while the morphologies and crystalline dimensions may exhibit differences from traditional pathways as the microwave allows rapid heat flow instead of convection as in traditional heating. SEs may be processed by microwave to reduce time and energy consumption, specifically for SEs prepared through wet synthesis [94, 95], also for the thermal treatment step [96, 97]. The synthesis of Li‐rich layered oxide cathode materials also benefits from the microwave, as processing at higher temperatures than co‐precipitation methods, microwave significantly boosts the reaction time within this process [98, 99]. The crystallization differences can be beneficial as the altered lattice parameters, crystalline sizes, and orientations [100] may enhance the ionic conductivity, while the lack of defects on SEs, CAMs, and the interlayers can prolong the lifetime of the cell [101, 102, 103].

**FIGURE 8:**
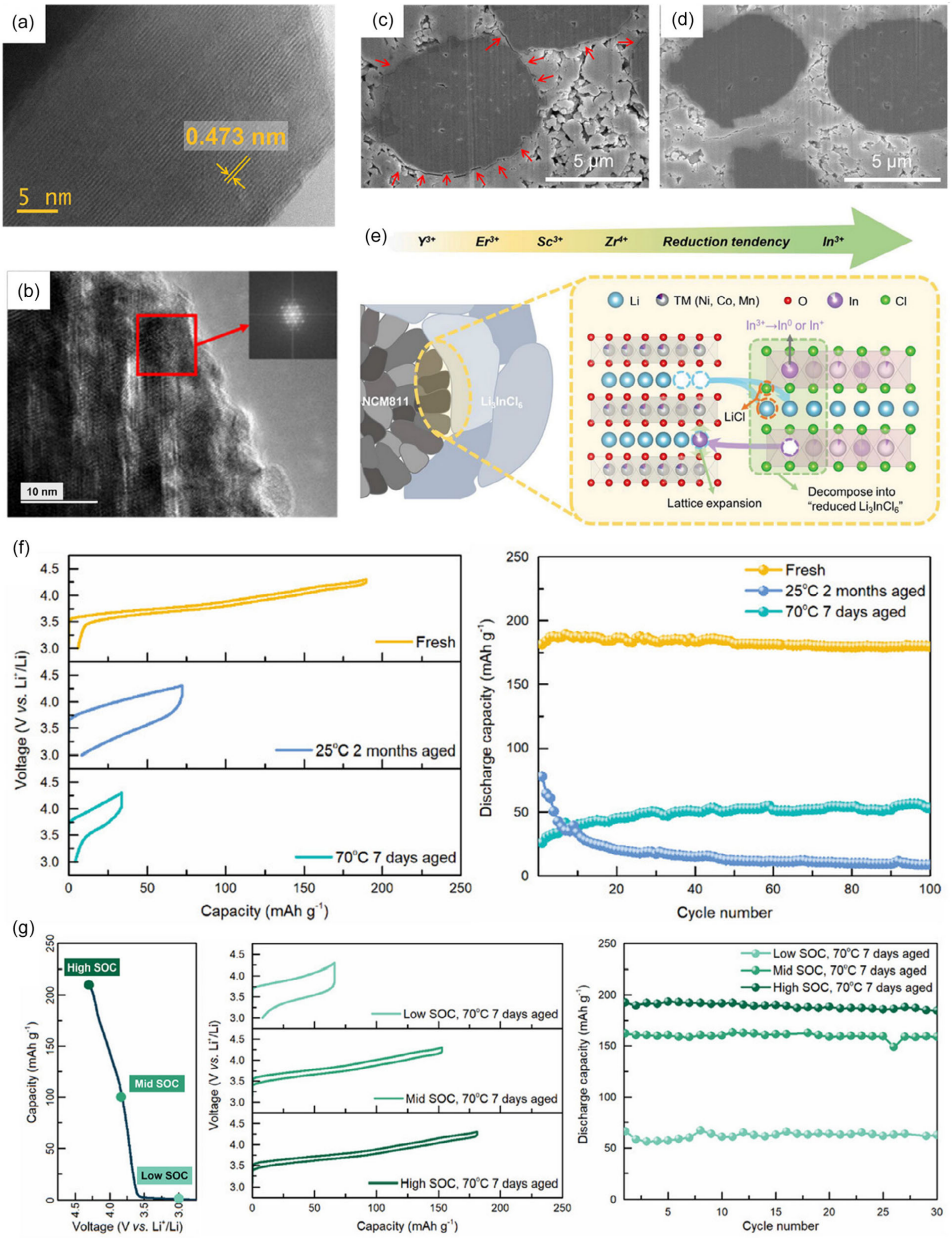
HSE and Cathode interfaces. (a) TEM image of layered oxide cathode [105]; (b) TEM image of pristine Li‐rich Mn (LRM)/LIC after 100 cycles, adapted from [106]; Cross‐sectional SEM images of (c) LNMO/LPSCl and (d) LNMO/LYC composites, both prior to charging, adapted from [107]; (e) Proposed mechanism of the interfacial degradation during aging, the susceptibility of LIC to reduction induces the intermixing of lithium and indium at the interface, deteriorating the transport properties, adapted from [108]; (f) Charge–discharge profiles of the second cycle of NMC811‐LIC SSBs fabricated using composite cathodes in fresh, 25°C 2 months aged, and 70°C 7 days aged conditions, and the capacity retention (at 20 mA g^−1^) of the cells employing composite cathodes aged under different aging conditions; (g) Charge profile of NMC811‐LIC SSB and selected state of charge (SOC) levels for 70°C 7 days aging experiments, the charge–discharge profiles of the second cycle of the SSBs aged at each SOC level, and the capacity retention (at 20 mA g^−1^) of the cells aged at each SOC level; (f) and (g) adapted from [108].

Though sulfide SEs and HSEs are considered soft materials compared with garnet SEs, the contact issue between SE and CAM particles within catholytes remains a key problem [104]. The loss of contact will result in significantly higher interfacial resistance and decay in capacity performance. High pressure is usually applied to compact the catholytes used in laboratory‐scale all‐solid‐state batteries to minimize the internal voids during fabrication (Figure 8c,d), and it plays a key role in the composite porosity [109]. Meanwhile, although the stacking pressure required to maintain the contact between catholyte and SEs is significantly lower than the fabrication pressure, neither is practical for commercial applications. It is shown that sulfide SEs and HSEs prior to compaction can have different porosity, where HSE/CAM catholytes have fewer voids compared with sulfide SEs [107]. Plus, CAMs may exhibit volume changes during their lithiation and delithiation processes, which may create voids after their expansion and shrinkage. The loss of contact after shrinkage can also lead to localized strains on CAMs, which may lead to accelerated structural degradation and performance deterioration [110, 111, 112, 113].

**FIGURE 9:**
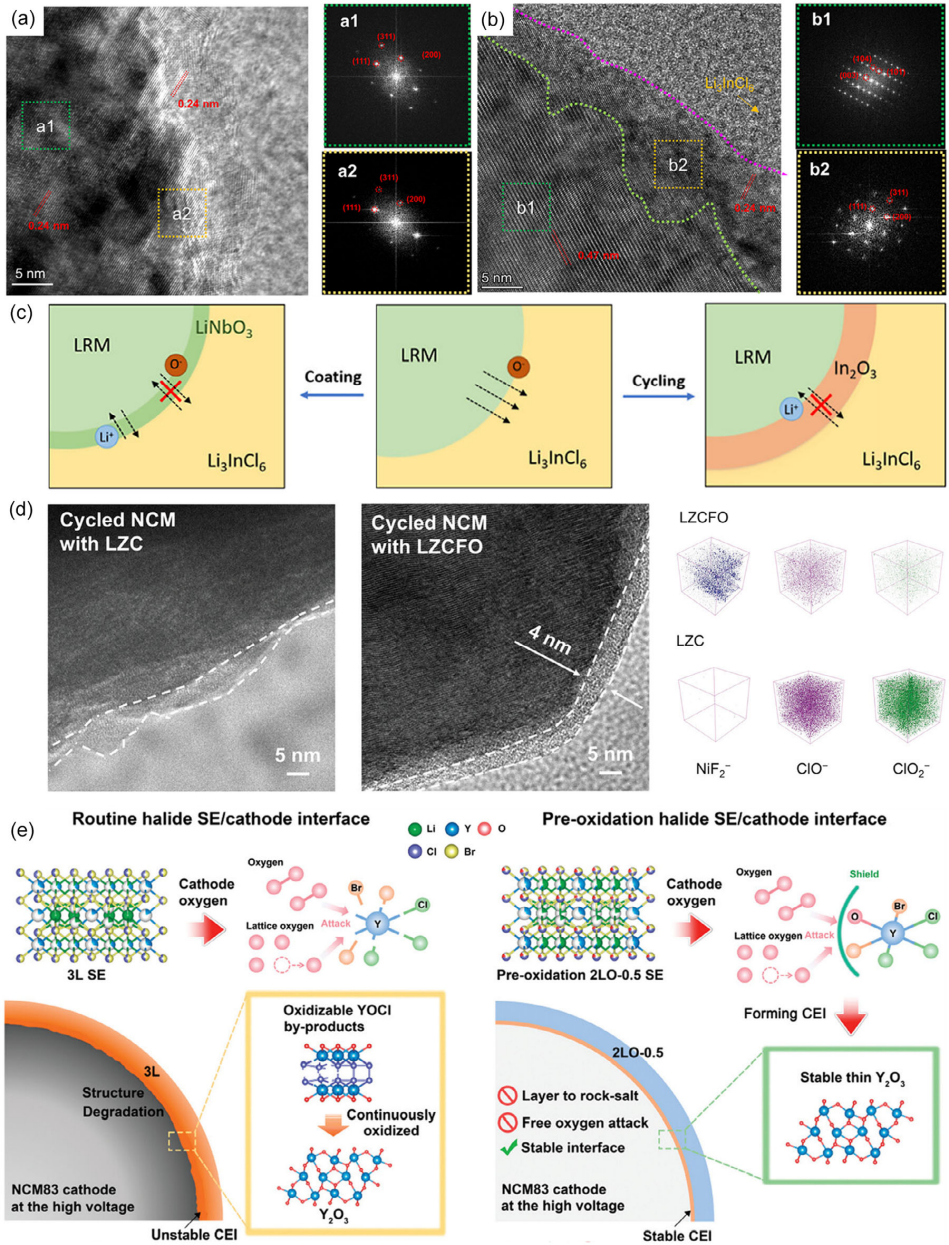
HR‐TEM images of (a) NMC and (b) 10% LIC@NMC after 100 cycles with LPSCl SE and the corresponding fast fourier transform maps in the selected regions, adapted from [121]; (c) LiNbO_3_ surface coating of Li‐rich Mn‐based (LRM) cathode acts as interfacial stabilizer between cathode and HSE, adapted from [106]; (d) TEM images of cycled NMC955 with Li_2_ZrCl_6_ (LZC) and Li_2.5_ZrCl_5_F_0.5_O_0.5_ (LZCFO), 3D views of NiF_2_‐, ClO‐, and ClO_2_‐ from ToF‐SIMS analysis, adapted from [89]; (e) Schematic illustrations of the CEI evolution in Li_3_YCl_3_Br_3_ (3L) and Li_2_YCl_2.5_Br_1.5_O_0.5_ (2LO‐0.5) solid‐state batteries, adapted from [122].

High‐voltage cathodes such as Li‐rich layered oxides can release oxygen during the early cycles or when overcharged [114, 115, 116]. For sulfide‐based SEs, aside from interfacial resistance caused by the resistive lithium‐deficient space‐charge layer [63, 117, 118, 119], the released oxygen can lead to degradation of thiophosphates, forming CEI consisting of PO_*x*_^*m*‐^ and MS_*y*_ reducing the ionic conductivity at the interfaces [65], while the high potential can also lead to the decomposition of the electrolyte, releasing Li_2_S, LiCl, and polysulfides [117, 120]. In the case of HSEs, the oxygen may lead to the formation of metal oxides or oxyhalides (MO_*x*_ or MO_*y*_X_*z*_) [79], essentially increasing the interfacial impedance. Plus, the diffusion of lithium and HSE center metal ions allows their exchange at the CAM/HSE interface, which can lead to the reduction of metal elements within HSEs over time and degrade the HSE (Figure 8e) [108], posing challenge for real‐world applications. Therefore, surface coating strategies are developed to address such issues on both CAMs and SEs, especially for composite catholytes. The ideal properties of such layers should be high ion conductivity and low electron conductivity, chemically stable with both SEs and CAMs. LiNbO_3_ (Figure 9c) and LiTaO_3_ are some examples to be coated on CAM particles, which are found to be effectively reducing interfacial resistance with sulfide SEs [123, 124, 125], though the exfoliation of such layer can lead to the release of oxygen and decomposition of CAM [65], and degradation of LiNbO_3_ can take place under high voltages and release oxygen [120]. The oxide‐coated CAMs are also shown to benefit their compatibility with HSEs, as the oxidation of HSEs is alleviated [107]. HSEs can be applied to CAM surfaces acting as an interfacial layer between sulfide SEs [121] (Figure 9a), while a mixture of HSE and sulfide SE shows noticeable capacity degradation after aging due to the loss of contact between CAM and SE within the catholyte [126]. Preoxidation of HSE (Figure 9e) has been examined to mitigate the uncontrolled formation of MO_*x*_ or MO_*y*_X_*z*_ from CAM‐released oxygen, stabilizing the CEI [122]. Overall, the interlayer between CAM and SEs plays a key role in chemical and mechanical compatibility, critical for the commercialization and prolonged operation of ASSLBs.

To distill the design landscape into a comparative framework, Table 1 provides a quantitative side‐by‐side comparison of representative strategies discussed in this review, including CCD, impedance evolution, cycling durability, and processing considerations. This comparison highlights the practical trade‐offs among electrochemical stability, mechanical compliance, and scalability that govern real‐world interlayer design.

**TABLE 1:** Quantitative and Qualitative comparison of representative interlayer strategies for sulfide‐ and halide‐based SEs in ASSLBs.

Interface/System	Interlayer strategy	Processing route	Electrochemical impact	Cost/Scalability	Ref.
Li/sulfide SE	In situ LiF–Li_3_N composite interphase formed by reacting pentafluorobenzamide with Li metal	In situ chemical reaction on Li	CCD increased to 3.25 mA cm^−2^ (LGPS) and 1.25 mA cm^−2^ (LPSCl), stable cycling 9000 h at 0.1 mA cm^−2^ with 0.1 mAh cm^−2^	Simple chemistry, scalable in principle	[43]
Li/sulfide SE	Thin Al_2_O_3_ coatings on LPSCl particles	ALD	CCD increased from 0.6 to 0.8 mA cm^−2^ after coating, Li/Li symmetrical cells with coated LPSCl had nearly two times lower impedance and sustained ≥150 cycles at 0.5 mA cm^−2^ compared to 115 cycles without coating	High quality/conformal, scalability may be limited by ALD throughput/cost	[45]
Li/sulfide SE membrane	Composite superhydrophobic Li^+^‐conducting protective layer: F‐POS@LATP on LPSCl membrane	Spray‐coating	LTO/F‐POS@LATP/LPSCl/F‐POS@LATP/LNO@LCO full cell had reversible capacity 147.3 mAh g^−1^, stable cycling up to 80 cycles, total impedance in symmetrical cell increases from 5.32 Ω (bare) to 9.95 –14.2 Ω with thicker coating	Spray‐coating is compatible with large‐area processing, strong manufacturability relevance	[47]
Li/sulfide SE	Anode‐architecture approach: Ag–C composite anodes	Electrode architecture	Full cells cycled stably for 1000 cycles (0.5 C) at high areal capacities of 6.8 mAh cm^−2^	Potentially scalable	[16]
Cathode/sulfide SE	ZrO_2_ coating on NMC111	Sol–gel + ultrasonic irradiation	Full cells with ZrO_2_ coating had initial discharge capacity ∼ 115 mAh g^−1^, capacity retained over 50 cycles at 0.1 mA cm^−2^, whereas control cells had only 105 mAh g^−1^ initial capacity and higher capacity fading	Solution‐based coating, good scalability potential	[66]
Cathode/sulfide SE	LiNbO_3_ coating on NMC111	Coating	Oxygen release from cathode is suppressed, LTO/LiNbO_3_@NMC cells had good capacity retention for 500 cycles	Widely used, scalability depends on coating method	[65]
Cathode/sulfide SE	Li_2_CO_3_/LiNbO_3_ composite coatings	Particulate coating	Reduced oxidized P/S species, improved cycling	Particle coating can be scalable	[68]
Cathode/sulfide SE	Amorphous LLSTO interlayer at NMC/LPSCl	Interlayer insertion	Markedly reduced polarization, exceptional long‐term cycling	Promising	[69]
Cathode/sulfide SE	PVDF–HFP‐based SPE interlayer at LFP/sulfide SE	Polymer interlayer insertion	Li/LFP ASSLBs showed stable cycling for 50 cycles at 0.1 C under zero external stack pressure	Polymers are scalable and mechanically compliant	[70]
Li/halide SE	LPSCl interlayer between Li and LYC	Powder stacking/cold pressing	Symmetric cell (at 0.2 mA cm^−2^) ∼100 mV for > 1000 h with LPSCl interlayer, Control: ∼1500 mV within 60 h	Simple powder processing, compatible with conventional pellet fabrication	[76]
Li/halide SE	PI_3_ interlayer → Li_6_PI_3_ formed in situ (for Li_3_YbCl_6_, etc.)	Interlayer that converts upon contact with Li	Reduced interfacial resistance and increased CCD	Likely scalable	[82]
Li/halide SE	Ag interlayer → Li–Ag alloy buffer	Cold pressing	CCD 1.3 mA cm^−2^, symmetric cell stable up to 500 h at 0.1 mA cm^−2^	Simple and economical, scalable via pressing	[84]
Li/halide SE	β‐Li_3_N interlayer with Li_2_ZrCl_6_	Ball‐milling + annealing to form phases	Impedance reduced 1929 Ω → ∼400 Ω, overpotential reduced 250 mV → ∼50 mV, stable ≥300 h at 0.1 mA cm^−2^	Commercial Li_3_N, scalable powders/anneal	[85]
Li/halide SE	LiF‐rich SEI + bilayer electrolyte (LIC/LPSCl)	Bilayer electrolyte + preformed LiF‐rich SEI	Full cell with LCO: >85% capacity retention after 100 cycles at 0.2 C	Practical concept, bilayer adds complexity	[86]
Li/halide SE	Fluoride‐coated halide interlayer: LFZ@LZC (LiF–ZrF_4_ shell)	In situ gas–solid fluorination	Symmetrical cell cycling > 800 h at 0.1 mA cm^−2^ and 0.1 mAh cm^−2^, electronic conductivity lowered ∼one order of magnitude	Promising, fluorination process needs scale‐up consideration	[83]
Cathode/halide SE	High‐valent metal doping in HSE catholyte: Li_2.6_In_0.8_Ta_0.2_Cl_6_ + NMC811	Catholyte composition tuning	70% capacity retention after 950 cycles at 1.39 mA cm^−2^, voltage window 3.0–4.4 V vs Li/Li^+^	Strong durability, composition tuning scalable if synthesis is scalable	[91]

Notably, inorganic ceramic interlayers generally demonstrate higher CCD enhancement and stronger suppression of interfacial impedance growth, whereas polymer‐based interlayers provide superior mechanical compliance and low‐pressure stability. Composite and fluoride‐derived interlayers exhibit a more balanced performance, combining moderate impedance control with improved scalability. As summarized in Table 1, these comparative metrics clarify the practical trade‐offs among electrochemical robustness, mechanical adaptability, and manufacturing compatibility. These quantitative comparisons indicate that optimal interlayer selection is application‐specific, depending on whether high‐rate capability, low‐stack‐pressure operation, or manufacturability is prioritized.

## 6 Conclusions and Outlook

As highlighted throughout this review and quantitatively summarized in Table 1, the ultimate performance of sulfide‐ and halide‐based ASSLBs is dictated not by bulk ionic conductivity alone, but by the stability and functionality of electrode–electrolyte interfaces. Sulfide and halide SEs have emerged as leading candidates for high‐energy‐density ASSLBs due to their exceptional ionic conductivities, low grain‐boundary resistance, and favorable mechanical deformability; however, their practical implementation continues to be constrained by severe interfacial instabilities with both Li‐metal anodes and high‐voltage oxide cathodes. Interlayer engineering, therefore, emerges as a unifying and application‐driven strategy to address these challenges by regulating interfacial chemistry, mitigating space‐charge effects, homogenizing Li^+^ transport, and stabilizing electrochemically and mechanically robust interphases under high current densities. This review has systematically recapitulated recent progress in interlayer‐engineering approaches across sulfide‐ and halide‐based ASSLB systems and distilled unified design principles linking electrochemical stability, ionic transport, and mechanical compliance across both anode and cathode interfaces.

Looking forward, the translation of interlayer‐enabled ASSLBs from laboratory‐scale demonstrations to practical devices requires a clear roadmap spanning several interdependent dimensions. The comparative analysis presented in Table 1 underscores that no single interlayer chemistry simultaneously optimizes CCD, impedance suppression, mechanical compliance, and scalability, reinforcing the need for application‐specific design frameworks. First, precise control of interface chemistry is essential, with interlayers designed either to form electronically insulating yet ionically conductive passivation layers or to enable controlled, self‐limiting mixed‐conducting interphases, depending on electrolyte chemistry and operating conditions. Second, mechanical and chemo‐mechanical adaptability must be prioritized through thin, conformal, and compliant interlayers capable of accommodating volume changes, contact loss, and stack‐pressure relaxation during long‐term cycling under low external pressures (<2 MPa). Third, future studies must move beyond pellet‐based testing toward cell‐relevant performance metrics, including high cathode areal loadings (>3 mAh cm^−2^), lean Li excess, limited stack pressure, and extended cycling in pouch‐type architectures.

Scalability and manufacturability must be treated as primary design constraints rather than secondary considerations. Several key industrial bottlenecks currently limit large‐scale implementation of interlayer engineering. One major challenge lies in precise thickness control of ultrathin interlayers, which often require nanometer‐to‐submicrometer precision to balance electronic insulation and minimal additional area‐specific resistance (ASR). Although ALD offers excellent conformality and thickness precision, its inherently low throughput, high capital cost, and limited m^2^‐scale processing speed present significant scalability limitations for large‐area electrode manufacturing. Emerging alternatives such as sol–gel coatings, slurry‐integrated interlayers, vapor‐phase fluorination, and solution‐based deposition provide improved scalability, yet further optimization is needed to ensure uniform thickness and defect‐free coverage at industrial scale.

Another critical challenge is achieving large‐area uniform coating across composite cathodes and solid electrolyte sheets. In practical pouch or prismatic cells, electrode areas extend to hundreds of square centimeters, where pinholes, thickness gradients, or incomplete surface coverage can lead to localized current concentration and premature failure. Roll‐to‐roll compatible coating strategies, spray deposition, and hybrid catholyte‐integrated interlayers show promise; however, maintaining conformal coverage within porous cathode architectures and complex particle networks remains nontrivial. Air and moisture stability further complicates manufacturing integration, particularly for sulfide and halide SEs that are sensitive to hydrolysis and surface degradation. H_2_S evolution, formation of oxyhalides, and surface contamination during material transfer between coating and assembly steps pose safety and reliability concerns under realistic dry‐room conditions. Strategies such as fluorination, compositional doping, pre‐formed artificial interphases, and air‐stable halide formulations represent promising directions, yet systematic evaluation under ppm‐level moisture environments relevant to industrial production is still required.

Finally, interlayers must maintain mechanical integrity and interfacial contact under practical low stack pressures (<2 MPa), as high external pressures commonly used in laboratory‐scale demonstrations are not viable in commercial pouch‐cell configurations. Designing interlayers that combine chemical robustness with mechanical compliance and long‐term structural stability will be essential for real‐world deployment.

Collectively, these directions define a rational framework for interlayer design that bridges fundamental interfacial chemistry with realistic cell‐level requirements, integrating interfacial energetics, ion–electron decoupling, mechanical robustness, and manufacturing integration, thereby accelerating the development of next‐generation high‐performance ASSLBs.

## Author Contributions

**Ruosi Qiao**: writing ‐ review & editing, software. **Madan Bahadur Saud**: conceptualization, software, formal analysis, investigation, writing – review & editing, resources, writing – original draft. **Hansheng Li**: conceptualization, investigation, writing – original draft, writing – review & editing, visualization, resources, software. **Yeqing Wang**: writing – review & editing, software, supervision. **M. Bilal Faheem**: writing – original draft, writing – review & editing, validation, supervision, software. **Quinn Qiao**: supervision, resources, funding acquisition, writing – original draft, writing – review & editing, investigation, software, project administration.

## Funding

This study was supported by NSF IUCRC Center for Solid‐State Electric Power Storage (CEPS) (2052611) and NSF Energy Storage Engine in Upstate New York (2315695).

## Conflicts of Interest

The authors declare no conflicts of interest.

## Data Availability

Data sharing not applicable to this article, as no datasets were generated or analyzed during the current study.
